# Supplementary material for: Gain-of-function mutation in TASK-4 channels and severe cardiac conduction disorder
Source: EMBO Mol Med. 2014 Jun 27;6(7):937–51. doi: 10.15252/emmm.201303783 (PMC4119356; doi:10.15252/emmm.201303783)
Supplement: Supplementary file 1 — Supplementary Figure S1 [file emmm0006-0937-SD1.pdf]

Supplementary Figure S1

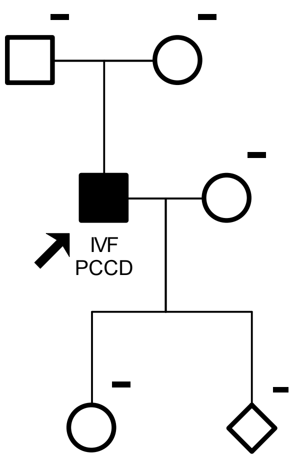

Supplementary Figure S1
Pedigree of family 10192. Men are denoted by squares, women by circles and family members with unknown sex by a rhombus. A – symbol indicates absence of DNA for genetic testing. A filled symbol represents a family member with a documented clinical phenotype of idiopathic ventricular fibrillation (IVF) and progressive cardiac conduction disorder (PCCD). The proband is indicated by an arrow.
