# Supplementary material for: Gain-of-function mutation in TASK-4 channels and severe cardiac conduction disorder
Source: EMBO Mol Med. 2014 Jun 27;6(7):937–51. doi: 10.15252/emmm.201303783 (PMC4119356; doi:10.15252/emmm.201303783)
Supplement: Supplementary file 2 — Supplementary Figure S2 [file emmm0006-0937-SD2.pdf]

Supplementary Figure S2

nucleotide	protein
**synonymous**	
c.339C>T	p.Thr113Thr
c.435C>T	p.Leu145Leu
c.591G>A	p.Arg197Arg
c.540G>A	p.Ala180Ala
c.735G>A	p.Val245Val
c.993C>T	p.Asp331Asp
**non-synonymous**	
c.61A>G	p.Ser21Gly
c.181G>A	p.Glu61Lys
c.265G>A	p.Ala89Thr
c.923A>G	p.Tyr308Cys
**in-frame deletion**	
c.324_326del	p.Phe109del
**intronic SNVs**	
c.353-16C>A	-
c.238-11C>G	-
c.1-23G>C	-

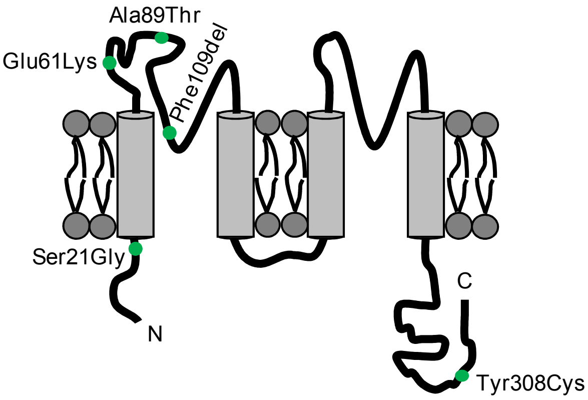

Supplementary Figure S2
Polymorphic sites in the *KCNK17* gene. In the patient cohort, a total of 14 polymorphic nucleotide sites were identified in *KCNK17*. TASK-4 α-subunit illustrating the localization of amino-acid exchanges (in green). All nucleotide variants were not considered as disease-causing because of benign pathogenicity prediction.
