# Supplementary material for: Gain-of-function mutation in TASK-4 channels and severe cardiac conduction disorder
Source: EMBO Mol Med. 2014 Jun 27;6(7):937–51. doi: 10.15252/emmm.201303783 (PMC4119356; doi:10.15252/emmm.201303783)
Supplement: Supplementary file 3 — Supplementary Figure S3 [file emmm0006-0937-SD3.pdf]

# Supplementary Figure S3

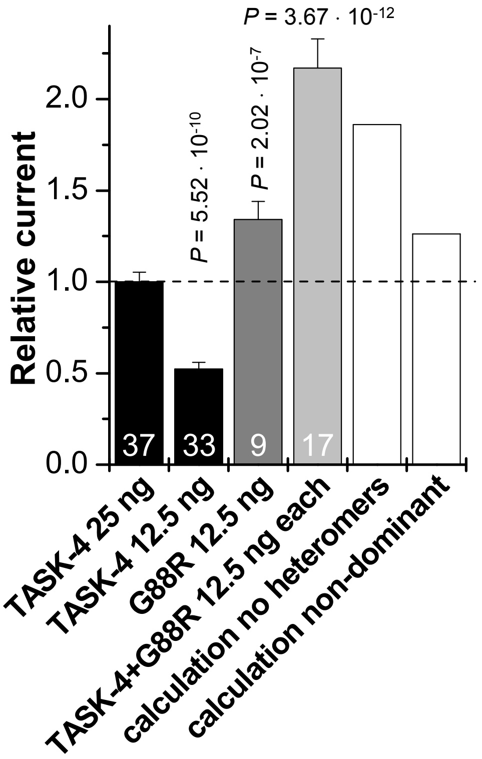


**Supplementary Figure S3**
Gain-of-function by G88R is conferred to heteromeric channels composed of wild-type and mutant G88R subunits. Experiments are performed in a linear range, as injection of twice the amount of TASK-4 cRNA into *Xenopus* oocytes leads to a doubling of the current amplitude. Injection of 12.5 ng G88R leads to a 2.58 ± 0.2 fold increase in current amplitude compared to injection of 12.5 ng wild-type cRNA. This is a similar gain-of-function, as we have observed, when using 25 ng of cRNA for the constructs (Fig. 6B). Co-expression of 12.5 ng wild-type TASK-4 with 12.5 ng of G88R leads to a pronounced current increase, which is bigger than adding the amplitudes for both the individual constructs (calculation no heteromers). Note that such an additive behavior would only occur if the channels would not form heteromers and express as separate homomeric channels. However, there is no evidence that the G88R mutant should fail to form heteromeric channels with wild-type TASK-4. Most importantly, after co-expression with wild-type and assuming a normal assembly, only 16.67 % of the channels would have two G88R subunits (Fig. 6C). Thus, if the gain-of-function would not be conferred to heteromeric channels with wild-type subunits, only 16.67 % of the dimeric channels would show a gain-of-function. The resulting current would be formed by 16.67 % of the G88R amplitude plus 83.33 % of wild-type amplitude. Calculating the expected current when only the channels with two G88R subunits have a gain-of-function (calculation non-dominant) shows that the observed strong current increase by co-expression can only be explained if heteromeric channels of wild-type and G88R subunits also have a gain-of-function. Data are provided as mean ± S.E.M.. *P*-values calculated in unpaired Student’s t-test are indicated. Numbers of independent experiments are indicated within the bars.
