# Supplementary material for: Gain-of-function mutation in TASK-4 channels and severe cardiac conduction disorder
Source: EMBO Mol Med. 2014 Jun 27;6(7):937–51. doi: 10.15252/emmm.201303783 (PMC4119356; doi:10.15252/emmm.201303783)
Supplement: Supplementary file 4 — Supplementary Table S1 [file emmm0006-0937-SD4.pdf]

Supplementary Table S1

Heterozygous nucleotide variants from CARDIO panel obtained by WES present in genome databases (EVS, dbSNP, Ensembl Gene Browser).

Chr., Chromosome.

Chr.	Gene	Chromosomal change g.	Transcript	Nucleotide change c.	Amino acid change p.	Reference genome database	Annotation	MAF (%)
1	*CLCNKB*	16378000 A>G	NM_000085.4	1255A>G	Ile419Val	EVS	rs6650119	13.06
1	*GJA4*	35260769 C>T	NM_002060.2	955C>T	Pro319Ser	EVS	rs1764391	37.81
1	*NEXN*	78392446 G>A	NM_144573.3	733G>A	Gly245Arg	EVS	rs1166698	15.78
1	*CLCA2*	86891073 G>A	NM_006536.5	238G>A	Val80Ile	EVS	rs11580625	3.07
1	*CLCA1*	86952324 A>G	NM_001285.3	1070A>G	Asn357Ser	EVS	rs2734705	13.08
1	*CLCA1*	86959173 T>C	NM_001285.3	1571T>C	Met524Thr	EVS	rs2791494	19.83
1	*KCNA10*	111060752 C>T	NM_005549.2	658C>A	Val1536Met	EVS	rs34970857	6.00
1	*CASQ2*	116310967 T>C	NM_001232.3	196A>G	Thr66Ala	EVS	rs4074536	33.94
1	*ATP1A4*	160134056 G>A	NM_144699.3	889G>A	Glu297Lys	EVS	rs17368402	8.34
1	*KCNH1*	210857116 C>A	NM_172362.2	2477G>T	Gly826Val	EVS	rs115026899	2.58
2	*APOB*	21225753 C>T	NM_000384.2	12541G>A	Glu4181Lys	EVS	rs1042031	16.61
2	*APOB*	21250914 G>A	NM_000384.2	1853C>T	Ala618Val	EVS	rs679899	36.55

2	*NEB*	152364589 G>A	NM_001164507.1	23381C>T	Ser7794Leu	EVS	rs41270201	2.06
2	*NEB*	152580815 C>G	NM_001164507.1	571G>C	Glu191Gln	EVS	rs35686968	1.89
2	*SCN7A*	167279922 C>A	NM_002976.3	2874G>T	Met958Ile	EVS	rs6738031	24.45
2	*SCN7A*	167334085 G>T	NM_002976.3	122C>A	Thr41Asn	EVS	rs7565062	29.83
2	*TTN*	179440163 C>G	NM_001267550.1	70696G>C	Gly23566Arg	EVS	rs55801134	1.14
2	*TTN*	179486223 C>T	NM_001267550.1	45328G>A	Asp15110Asn	EVS	rs17354992	0.667
3	*SCN5A*	38645420 T>C	NM_001099404.1	1673A>G	His558Arg	EVS	rs1805124	24.63
3	*SCN10A*	38764998 A>G	NM_006514.2	3275T>C	Leu1092Pro	EVS	rs12632942	22.00
3	*SCN10A*	38766675 A>G	NM_006514.2	3218T>C	Val1073Ala	EVS	rs6795970	29.75
3	*SCN10A*	38768300 T>C	NM_006514.2	2884A>G	Ile962Val	EVS	rs57326399	21.03
5	*ADRB2*	148206440 G>A	NM_000024.5	46G>A	Gly16Arg	EVS	rs1042713	41.49
6	*ITPR3*	33659472 C>G	NM_002224.3	7306C>G	Leu2436Val	EVS	rs2229642	40.84
6	*TRDN*	123699019 A>C	NM_006073.3	1211T>G	Val404Gly	EVS	rs28494009	15.87
6	*TRDN*	123869607 G>C	NM_006073.3	383C>G	Thr128Ser	EVS	rs9490809	45.88
6	*LAMA2*	129807629 C>T	NM_000426.3	7760C>T	Ala2587Val	EVS	rs2229848	36.14
6	*LAMA2*	129813053 A>G	NM_000426.3	7906A>G	Thr2636Ala	EVS	rs2244008	8.08
6	*SYNE1*	152443744 G>T	NM_182961.3	26221C>A	Leu8741Met	EVS	rs2295190	10.41
6	*SYNE1*	152453291 G>A	NM_182961.3	26060C>T	Thr8687Ile	EVS	rs35591210	5.44
6	*SYNE1*	152640110 G>A	NM_182961.3	16227C>T	Thr5426Met	EVS	rs2306914	2.00
6	*SYNE1*	152665261 C>A	NM_182961.3	12180G>T	Glu4060Asp	EVS	rs4645434	44.27

6	*SYNE1*	152746593 A>T	NM_182961.3	5190T>A	Asp1730Glu	EVS	rs111250109	4.82
6	*SYNE1*	152772264 A>G	NM_182961.3	3104T>C	Val1035Ala	EVS	rs214976	46.75
6	*SYNE1*	152777095 A>C	NM_182961.3	2653T>G	Leu885Val	EVS	rs17082709	6.34
7	*AKAP9*	91630620 G>T	NM_005751.4	1389G>T	Met463Ile	EVS	rs6964587	44.05
7	*AKAP9*	91652179_91652181dup	NM_005751.4	c.4004_4006dup	Lys1335_Leu1336 insGln	EVS	rs10644111	47.91
7	*AKAP9*	91712698 A>G	NM_005751.4	8375A>G	Asn2792Ser	EVS	rs6960867	35.61
8	*KCNQ3*	133175736 T>C	NM_004519.3	1241A>G	Glu414Gly	EVS	rs2303995	1.60
9	*ABCA1*	107562804 T>C	NM_005502.3	4760A>G	Lys1587Arg	EVS	rs2230808	41.5
9	*ABCA1*	107620867 C>T	NM_005502.3	656G>A	Arg219Lys	EVS	rs2230806	39.15
10	*MYPN*	69926334 C>G	NM_001256267.1	1884C>G	Phe628Leu	EVS	rs10823148	39.6
10	*MYPN*	69933921 G>A	NM_001256267.1	2072G>A	Ser691Asn	EVS	rs10997975	39.24
10	*MYPN*	69933969 G>A	NM_001256267.1	2120G>A	Ser707Asn	EVS	rs7916821	39.23
10	*MYPN*	69934258 C>G	NM_001256267.1	2409C>G	Ser803Arg	EVS	rs3814182	47.7
10	*MYPN*	69959242 C>A	NM_001256267.1	3403C>A	Pro1135Thr	EVS	rs7079481	40.59
10	*BAG3*	121429633 T>C	NM_004281.3	451T>C	Cys151Arg	EVS	rs2234962	15.11
14	*SYNE2*	64537498 A>C	NM_182914.2	10567A>C	Lys3523Gln	EVS	rs35203186	2.65
14	*SYNE2*	64557734 A>C	NM_182914.2	11944A>C	Asn3982His	EVS	rs10137972	8.63
14	*KCNK10*	88651962 C>T	NM_138317.2	1549G>A	Ala517Thr	EVS	rs17762463	20.51
14	*KCNK13*	90651033 G>A	NM_022054.2	913G>A	Gly305Arg	EVS	rs3814848	13.24

15	*RYR3*	33873751 G>A	NM_001036.3	1480G>A	Val494Ile	EVS	rs2077268	15.73
15	*RYR3*	33954652 C>T	NM_001036.3	4921C>T	Arg1641Cys	EVS	rs4780144	13.19
15	*RYR3*	34137086_34137088del	NM_001036.3	13320_13322del	Glu4441del	EVS	rs149087920	13.51
15	*MEF2A*	100252710	NM_005587.2	1234-1236del	Gln412del	EVS	rs72198683	29.32
16	*CACNA1H*	1252441 T>C	NM_021098.2	1991T>C	Val664Ala	EVS	rs4984636	17.98
16	*CACNA1H*	1269003 A>G	NM_021098.2	5921A>G	Glu1974Gly	EVS	rs3751886	4.34
16	*CACNA1H*	1270585 G>A	NM_021098.2	6653G>A	Arg2218His	EVS	rs56885166	0.20
16	*CLCN7*	1502857 C>T	NM_001287.5	1252G>A	Val418Met	EVS	rs12926089	12.40
16	*MYH11*	15820863 C>T	NM_001040114.1	3721G>T	Ala1241Thr	EVS	rs16967494	20.68
17	*KCNJ12*	21318698 C>T	NM_021012.4	44C>T	Ser15Leu	Ensembl	rs1657738	50.00
17	*KCNJ12*	21318782 G>A	NM_021012.4	128G>A	Arg43His	Ensembl	rs78117732	50.00
17	*KCNJ12*	21318821 A>C	NM_021012.4	167A>C	Glu56Ala	EVS	rs1714865	34.15
17	*KCNJ12*	21318867 G>A	NM_021012.4	213G>A	Met71Ile	Ensembl	rs73979893	50.00
17	*KCNJ12*	21319121 C>T	NM_021012.4	467C>T	Pro156Leu	Ensembl	rs1714864	50.00
17	*KCNJ12*	21319171 G>A	NM_021012.4	517G>A	Asp173Asn	Ensembl	rs73313922	multiple observations
17	*KCNJ12*	21319208 C>T	NM_021012.4	554C>T	Ala185Val	Ensembl	rs73979896	multiple observations
17	*KCNJ12*	21319230 G>C	NM_021012.4	576G>C	Gln192His	Ensembl	rs1657742	multiple observations

17	*KCNJ12*	21319285 C>T	NM_021012.4	631C>T	Leu211Phe	Ensembl	rs72846667	multiple observations
17	*KCNJ12*	21319682 C>T	NM_021012.4	1028C>T	Ser343Leu	Ensembl	rs80203231	multiple observations
17	*JUP*	39912145 T>A	NM_002230.2	2089T>A	Met697Leu	EVS	rs1126821	30.30
18	*DSC2*	28648000_28648001dup	NM_024422.3	c.2686_2687dup	Ala897Lys*4	EVS	unknown	0.96
18	*DSG2*	29122799 G>A	NM_001943.3	2318G>A	Arg773Lys	EVS	rs2278792	19.73
18	*TTR*	29172865 G>A	NM_000371.3	76G>A	Gly26Ser	EVS	rs1800458	5.74
19	*GDF1*	18980172 G>A	NM_001492.4	353C>T	Ala118Val	dbSNP	rs4808863	28.4
19	*RYR1*	38976655 C>T	NM_000540.2	5360C>T	Pro1787Leu	EVS	rs34934920	1.67
19	*RYR1*	38983180 G>T	NM_000540.2	6178G>T	Gly2060Cys	EVS	rs35364374	5.14
20	*KCNK15*	43379264 A>C	NM_022358.3	778A>C	Thr260Pro	EVS	rs6073538	46.29
20	*KCNK15*	43379454 T>C	NM_022358.3	968T>C	Leu323Pro	EVS	rs13042905	40.00
20	*KCNQ2*	62038277 T>G	NM_172107.2	2339A>C	Asn780Thr	EVS	rs1801475	40.13
21	*KCNE1*	35821821 T>C	NM_000219.4	112A>G	Ser38Gly	EVS	rs1805127	33.81
21	*KCNJ15*	39671476 G>A	NM_002243.3	293G>A	Gly98Asp	EVS	rs2230033	40.29
